# Shaping Synapses by the Neural Extracellular Matrix

**DOI:** 10.3389/fnana.2018.00040

**Published:** 2018-05-15

**Authors:** Maura Ferrer-Ferrer, Alexander Dityatev

**Affiliations:** ^1^Molecular Neuroplasticity German Center for Neurodegenerative Diseases (DZNE), Magdeburg, Germany; ^2^Center for Behavioral Brain Sciences (CBBS), Magdeburg, Germany; ^3^Medical Faculty, Otto-von-Guericke University, Magdeburg, Germany

**Keywords:** perineuronal net, synaptogenesis, synaptic plasticity, proteolysis, disease

## Abstract

Accumulating data support the importance of interactions between pre- and postsynaptic neuronal elements with astroglial processes and extracellular matrix (ECM) for formation and plasticity of chemical synapses, and thus validate the concept of a tetrapartite synapse. Here we outline the major mechanisms driving: (i) synaptogenesis by secreted extracellular scaffolding molecules, like thrombospondins (TSPs), neuronal pentraxins (NPs) and cerebellins, which respectively promote presynaptic, postsynaptic differentiation or both; (ii) maturation of synapses via reelin and integrin ligands-mediated signaling; and (iii) regulation of synaptic plasticity by ECM-dependent control of induction and consolidation of new synaptic configurations. Particularly, we focused on potential importance of activity-dependent concerted activation of multiple extracellular proteases, such as ADAMTS4/5/15, MMP9 and neurotrypsin, for permissive and instructive events in synaptic remodeling through localized degradation of perisynaptic ECM and generation of proteolytic fragments as inducers of synaptic plasticity.

## Introduction

Synapses are considered as the functional units that enable information processing of the brain. A synapse is formed when a presynaptic button establishes a connection with a postsynaptic cell. However, research in the past years has evinced the active participation of astrocytes and their processes in pre- and postsynaptic functions, leading to the tripartite synapse concept (Araque et al., 1999). It is supported by an extensive number of studies highlighting the important role of astrocytes in the formation, maturation and elimination of synapses (Chung et al., 2015). For instance, Halassa et al. (2007) found that, on average, a single astrocyte enwraps four neuronal somata and contacts 300–600 neuronal dendrites. Moreover, cortical and hippocampal astrocytes occupy non-overlapping territories and can contact ~140,000 synapses (Bushong et al., 2002; Halassa et al., 2007). More recently, the concept of tripartite synapse was “upgraded” to the tetrapartite version (SYNAPSE 4.0) in the light of the evidenced role of extracellular matrix (ECM) as a fourth essential part of the synapse (Dityatev and Schachner, 2006; Dityatev et al., 2006; Faissner et al., 2010; Dityatev and Rusakov, 2011). Neural ECM is formed in an activity-dependent manner (Matthews et al., 2002; Brückner et al., 2003; Dityatev et al., 2007). ECM incorporates molecules secreted from both neurons and astrocytes, although even its highly condensed form of perineuronal nets (PNNs), can be formed in cultured cortical neurons in the absence of astrocytes (Miyata et al., 2005). Besides PNNs, ECM molecules are broadly expressed in the nervous tissue in a diffuse manner, as interstitial ECM, particularly in the perisynaptic space (Dityatev and Schachner, 2006).

**Figure 1:**
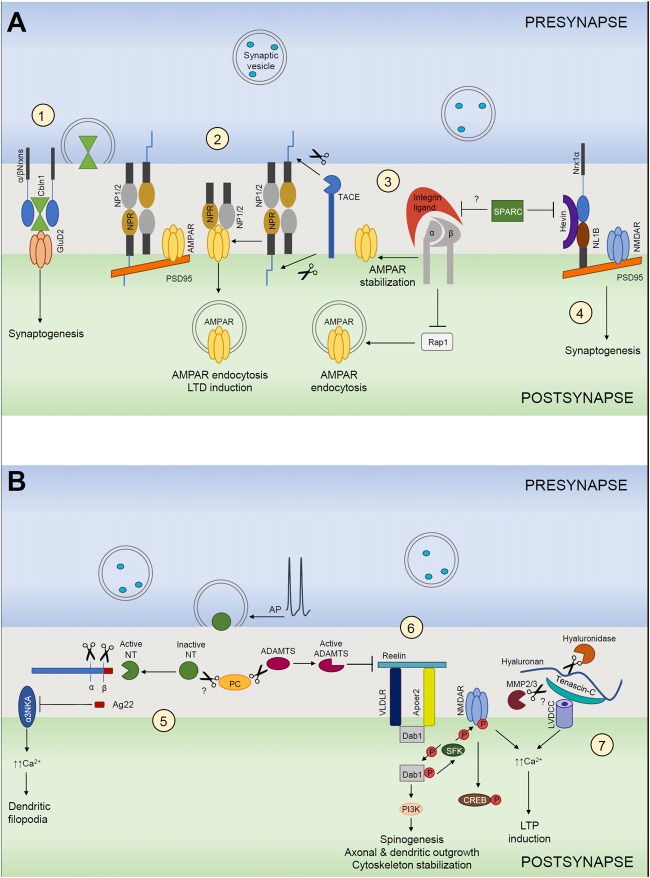
Extracellular matrix (ECM) in structural and functional synaptic plasticity. **(A)** (1) Cerebellin 1 (Cbln1) released from presynaptic neurons binds to its presynaptic α- and β-neurexins (α/βNrxn) receptors and directly recruits postsynaptic GluD2 located on postsynaptic neurons. The tripartite complex induces and maintains excitatory synapses. (2) Neuronal pentraxin 1 and 2 (NP1/2) and neuronal pentraxin receptor (NPR) co-aggregate with GluA4 subunit-containing AMPARs in neurons. Cleavage of NPR by TACE triggers AMPAR endocytosis during LTD. NP1/2 are secreted mainly from the presynaptic side, but where they bind to NPR remains unclear. (3) Integrins are involved in homeostatic synaptic scaling. Pharmacological blockade of action potentials leads to a “scaling up” of the AMPARs present at the synapse. This requires an increase of β3 integrins at the surface, which results in inhibition of the small GTPase Rap1, which normally stimulates the endocytic removal of GluA2-containing AMPARs from the cell surface. As a net outcome, synaptic expression of AMPARs is increased, resulting in larger excitatory postsynaptic currents. This mechanism is most likely negatively regulated by SPARC. (4) Hevin secreted from astrocytes induces synaptogenesis by bridging direct interaction-incompatible Neurexin-1α (Nrx1α) and Neuroligin-1B (NL1B) across the synaptic cleft. SPARC strongly inhibits Hevin-induced excitatory synaptogenesis *in vitro*. **(B)** (5) The neuronal serine protease neurotrypsin (NT) is stored in presynaptic terminals and secreted in an inactive form in response to presynaptic action potential (AP) firing. NT contains a pro-domain that inhibits its proteolytic activity. Its activation requires a zymogen activation presumably by a proprotein convertase (PC). Activated neurotrypsin cleaves agrin and yields a C-terminal 22-kDa fragment (Ag22), which inhibits the α3 subunit of the Na+/K+ ATPase (α3NKA) and induces dendritic filopodia formation. (6) Reelin signals through its receptors, very-low-density lipoprotein receptor (VLDLR) and apolipoprotein E receptor type 2 (APOER2), which interact with adaptor protein disabled 1 (Dab1). Activation of reelin signaling pathway results in cytoplasmic adaptor protein disabled 1 (Dab1) phosphorylation via the Src family of tyrosine kinases (SFK), activation of SFKs and tyrosine phosphorylation of NMDARs that increases receptor activity. This mechanism induces LTP and spinogenesis and it is negatively regulated by ADAMTS dependent reelin cleavage. (7) Hyaluronic acid and tenascin-C support the activity of neuronal L-type voltage-gated Ca^2+^ channels (LVGCCs). Ca^2+^ influx through NMDARs and LGCCs induces diverse forms of LTP.

In the last years, microglia emerged as another player targeting synaptic structures. Particularly, microglial processes appear adjacently to presynaptic boutons where they stay for a few minutes before retracting. These structural interactions are activity-dependent (Wake et al., 2009; Kettenmann et al., 2013) and may result in induction of filopodia formation (Miyamoto et al., 2016) or may lead to phagocytosis of existing synapses, i.e., “synaptic stripping”, by microglia (Trapp et al., 2007; Kettenmann et al., 2013). Thus, transiently, some synapses can have pentapartite structure, in which pre- and postsynaptic terminals are closely interacting with astrocytes, microglia and ECM. The purpose of this review is to outline the major ECM-dependent mechanisms driving synaptogenesis, maturation and activity-dependent remodeling of synapses in health and disease (Figure 1).

## Thrombospondins as Astrocyte-Secreted Proteins That Promote Presynaptic Differentiation

Thrombospondins (TSPs) are extracellular multimeric multidomain calcium-binding glycoproteins that function at the cell surface and bind different components of the ECM. In mammals, TSPs constitute a family with five members. TSP1 and TSP2, which evolved in vertebrates, exist as trimers, whereas the more ancient TSP3-TSP5 form pentamers (Adams, 2001). TSPs are major synaptogenic factors secreted by astrocytes (Christopherson et al., 2005; Eroglu et al., 2009). The addition of TSP1 and TSP2 to cultured neurons mimicked the astrocyte-conditioned medium-induced increase in synapse number. Similarly, removal of TSP from astrocyte-conditioned medium eliminated the majority of its synaptogenic activity. In line with this finding, TSP1/2 double knockout (KO) mice exhibited 30% decrease of cortical excitatory synapses. However, although electron microscopy revealed that TSP-induced synapses were ultrastructurally normal, whole-cell recordings revealed that these synapses were presynaptically active but postynaptically silent, lacking functional AMPA receptors (AMPAR; Christopherson et al., 2005). In agreement with playing a role in initiating synaptogenesis *in vivo*, TSP1 and TSP2 expression peaks coincide with the initiation of nascent synaptic contacts between dendrites and axons in the mouse brain (circa P5), but they decrease in the adult brain (Christopherson et al., 2005; Risher and Eroglu, 2012). Additionally, TSP4 is detected at the neuromuscular junction (NMJ), suggesting a possible role for TSP4 in synapse formation in the peripheral nervous system (Arber and Caroni, 1995). Inhibition of TSPs leads to defects in post-injury-induced structural plasticity of the developing barrel cortex (Eroglu et al., 2009). Similarly, upon injury in the spinal nerve, both TSP1 and TSP4 are upregulated in the spinal cord (Valder et al., 2003; Benton et al., 2008). TSP expression levels are also altered in the brain after stroke. TSP1 and TSP2 are significantly elevated after brain ischemia in rodents (Lin et al., 2003; Liauw et al., 2008). Although it was initially thought that TSP1 and TSP2 were contributing to the postischemic angiongenesis process, Liauw et al. (2008) observed no significant differences in blood vessel density between TSP1/2 KO mice and their WT littermates. More recently, TSP1 levels were found to be upregulated in plasma in human patients after stroke and moreover, higher TSP1 plasma concentrations correlated with unfavorable outcome of patients (Gao et al., 2015).

All five isoforms share the ability to induce synapse formation by interacting with the neuronal receptor α2δ-1 (Cacna2d1). More specifically, they bind the von Willebrand factor A domain of neuronal Cacna2d1 via their shared type 2 EGF-like repeats (Eroglu et al., 2009). Interestingly, this is the receptor for the anti-epileptic and analgesic drug Gabapentin. Gabapentin antagonizes TSPs binding to Cacna2d1 and inhibits excitatory synapse formation *in vitro* and *in vivo*, suggesting that gabapentin may act therapeutically by blocking new synapse formation without affecting previously formed synapses (Eroglu et al., 2009). Additionally to Cacna2d1, TSPs also interact with other cell-surface receptors and mediate other functions in the CNS (reviewed in Risher and Eroglu, 2012). TSP1 has also been shown to interact with the neuroligin 1 (NL1) extracellular domain *in vitro* and knocking down endogenous NL1 inhibited TSP1-induced excitatory synaptogenesis in cultured rat hippocampal neurons (Xu et al., 2010).

## Neuronal Pentraxins as Promoters of Postsynaptic Clustering

Pentraxins (PTXs) are a superfamily of multifunctional proteins characterized by a PTX domain. They are divided into short and long PTXs. NPs comprise neuronal pentraxin 1 (NP1), neuronal pentraxin 2 (NP2) and neuronal pentraxin receptor (NPR), which belong to the family of long PTXs (reviewed by Yuzaki, 2018). NP1 and NP2 are secreted and exist as multimeric complexes. Their relative ratio in the complex is dynamically dependent on the neuronal activity state and the developmental stage (Xu et al., 2003). NPR is a transmembrane protein but it can form heteropentamers with NP1 and NP2 and can be released from cell membranes when it is cleaved by the matrix metalloproteinase (MMP) tumor necrosis factor-α converting enzyme (TACE; Kirkpatrick et al., 2000; Cho et al., 2008; Figure 1A2). NP1, NP2 and NPR are broadly expressed in the hippocampus (CA3 and dentate gyrus), the cerebral cortex and the cerebellum (Schlimgen et al., 1995; Tsui et al., 1996; Dodds et al., 1997). NPs have been shown to associate with the AMPARs N-terminal domain via their PTX domains *in vitro* and *in vivo* (Figure 1A2). Moreover, overexpression of exogenous NP fragments induced clustering of postsynaptic AMPARs (O’Brien et al., 1999, 2002; Xu et al., 2003; Cho et al., 2008). NP2 has been found to concentrate at excitatory synapses on parvalbumin-expressing interneurons (PV-INs) and, notably, its synaptic accumulation depends on integrity of perisynaptic ECM of PNNs. Furthermore, activity-dependent changes in NP2 mediate coordinated changes in GluA4 AMPARs at excitatory synapses on PV-INs during epileptiform activity-driven homeostatic up-scaling of these synapses (Chang et al., 2010). Interestingly, a recent study has reported that post-mortem human Alzheimer’s Disease (AD) brains showed substantial reductions of NP2 and likewise reductions of GluA4. Moreover, the expression levels of NP2 have been found to be reduced in human CSF from AD subjects and to show robust correlation with cognitive performance and hippocampal volume in these patients (Xiao et al., 2017). Additionally, NPs are suggested to be involved in disorders with inhibition/excitation (I/E) imbalances such as schizophrenia, as it was reported that a combined loss of NP2 and NPR strongly reduced GluA4 expression leading to diminished excitation of PV-INs and impaired feed-forward inhibition *in vivo*. In agreement with these observations, the resulting I/E imbalance disrupted hippocampal rhythmogenesis, promoted epileptic activity and impaired hippocampal-dependent working memory (Pelkey et al., 2015).

## Molecules Cross-Linking Pre- and Postsynaptic Machineries

Early studies of synaptogenesis were pointing to the key role of trans-synaptic interactions between pre- and postsynaptically located cell adhesion molecules (CAMs), such as N-cadherins, for stabilization of pre- and postsynapses (Tanaka et al., 2000). Additionally, a concept was introduced that ECM molecules, such as heparan sulfate proteoglycans, may serve as extracellular scaffolds coordinating signaling through presynaptic receptors (FGFR1) and postsynaptic CAMs (polysialylated form of NCAM) to promote NMDA receptor (NMDAR) activity-dependent preferential formation and remodeling of synapses (Dityatev et al., 2004; Dityatev, 2006). More recent studies revealed several classes of extracellular scaffolds to be important for formation of excitatory synapses.

### Role of Hevin and SPARC in Synapse Formation

Hevin (also known as secreted protein acidic and rich in cysteine SPARC-like 1) and its homolog SPARC are astrocyte-secreted proteins that control synaptogenesis (Brekken and Sage, 2000; Kucukdereli et al., 2011). Hevin and SPARC expression *in vivo* peaks during the second and third postnatal weeks (Kucukdereli et al., 2011), a period that correlates with the peak of synaptogenesis. Interestingly, unlike TSP1–3, astrocytes persist to express both hevin and SPARC throughout adulthood. However, hevin expression levels remain very high, whereas SPARC expression is considerably reduced in the adult CNS (Eroglu, 2009). Hevin has been shown to localize at the excitatory synaptic clefts in the CNS (Johnston et al., 1990; Lively et al., 2007; Lively and Brown, 2008). Similar to TSPs, hevin induces synapse formation between cultured retinal ganglion cells (RGCs). However, unlike hevin, SPARC is not synaptogenic. Conversely, SPARC strongly inhibited hevin-induced excitatory synaptogenesis *in vitro*. In agreement with this finding, *Hevin*-null mice exhibited a significant impairment in the formation and maturation of synaptic connections in the superior colliculus (SC), with which RGCs make synapses. In contrast, SPARC-null mice showed increased RGC–SC synapse formation *in vivo*. Additionally, RGCs cocultured with SPARC and either TSP or hevin demonstrated that SPARC antagonized hevin’s synaptogenic activity but did not prevent TSP-induced synapse formation. Additionally, SPARC negatively regulates AMPARs recruitment to synapses, most likely via interactions with β3-integrins (Jones et al., 2011; Figure 1A3). Moreover, SPARC prevents maturation of cholinergic presynaptic terminals (Albrecht et al., 2012) and induces a cell-autonomous program of synapse elimination via retraction of axon terminals (López-Murcia et al., 2015). Similar to TSPs, hevin-induced synapses are ultrastructurally normal but postsynaptically silent (Kucukdereli et al., 2011). Risher et al. (2014), found that *Hevin*-null mice displayed a reduction in the number of thalamocortical synapses, accompanied by a transient increase of intracortical synapses. Interestingly, a recent study has shown that SPARC expression is selectively high in the hypothalamus and thalamus regions (Morel et al., 2017). Taken together, these findings suggest that hevin and SPARC may regulate thalamocortical synapse formation.

How does hevin induce synaptogenesis? It organizes pre- and postsynaptic specializations and induces thalamocortical synaptogenesis in the developing visual cortex by bridging neurexin-1α (NRX1α) and neuroligin-1B (NL1B), two isoforms that do not directly interact with each other (Singh et al., 2016; Figure 1A4). Interactions between presynaptic neurexins and postsynaptic neuroligins coordinate the formation of synaptic adhesions and are critical for the formation and maturation of synapses (Baudouin and Scheiffele, 2010). Thus, hevin acts as an essential organizer of both pre- and postsynaptic specializations and aligns them across the synapse. Mutations in neurexins and neuroligins are associated with several neurological disorders, including addiction, schizophrenia, depression and autism spectrum disorders (Südhof, 2008). Therefore, perturbations in the NRX1α-hevin-NL1B interaction could play a critical role in synaptic pathologies seen in these diseases. Similarly, hevin and SPARC showed altered expression patterns in a Fragile X Syndrome (FXS) mouse model caused by a deficiency in the fragile X mental retardation protein (FMRP). FMRP has been shown to regulate the translation of many mRNAs and its deficiency disturbs the composition of proteins important for dendritic spine and synapse development (Wallingford et al., 2017).

### Cerebellins: Another Bridge Between Pre- and Postsynaptic Structures

Cerebellins (Cblns) are secreted hexameric glycoproteins that belong to the C1q and tumor necrosis factor (TNF) superfamily (Kishore et al., 2004). Cerebellins function as trans-synaptic linkers as they form tripartite complexes with the presynaptic neurexins or “deleted in colorectal cancer” (DCC) and the postsynaptic delta-type glutamate (GluD1 and GluD2) receptors (Ito-Ishida et al., 2008; Matsuda et al., 2010; Uemura et al., 2010; Lee et al., 2012; Wei et al., 2012; Haddick et al., 2014). In vertebrates, there are four cerebellins (Cbln1–4) that are widely expressed throughout the brain (Miura et al., 2006; Wei et al., 2012; Cagle and Honig, 2014; Seigneur and Südhof, 2017). Cbln1 and Cbln3 are mainly expressed in cerebellar granular cells (GCs) and Cbln2 and Cbln4 are heterogeneously expressed in the forebrain (Pang et al., 2000; Miura et al., 2006). Cbln1, the most well studied isoform, is secreted from presynaptic terminals in GCs and has been shown to be essential for the establishment of parallel fiber (PF)-Purkinje cell synapses in the cerebellum (Hirai et al., 2005). Cbln1 acts as a bridge between presynaptic α- and β-neurexins on granule neurons and postsynaptic GluD2 on Purkinje cells (Matsuda et al., 2010; Uemura et al., 2010; Cheng et al., 2016; Elegheert et al., 2016), thereby stabilizing synaptic contacts (Figure 1A1). *Cbln1*-null mice showed synaptic loss in the cerebellum, ataxia and diminished motor learning (Hirai et al., 2005; Rong et al., 2012) as well as impaired cued and contextual fear memory (Otsuka et al., 2016). Interestingly, *Cbln1*-null mice showed a different phenotype in striatum. Conversely to cerebellum, *Cbln1*-deficient thalamic axons revealed an increase in synaptic spine density instead of a synapse loss (Kusnoor et al., 2010). Cbln2 has been reported to bind neurexins and induce presynaptic differentiation in cortical cultures (Joo et al., 2011). Cbln3 forms a heterohexamer with Cbln1 and binds to Purkinje cell dendrites in a similar manner as Cbln1 homomers (Pang et al., 2000; Matsuda et al., 2009). On the contrary, although Clbn4 is structurally similar to Cbln1–3, it was originally reported to bind only weakly to neurexins, but to bind with a high affinity to the netrin receptor “DCC” (Wei et al., 2012; Haddick et al., 2014). However, more recently, Cbln4 was found to bind to NRX1β and to form a stable complex with it (Zhong et al., 2017). Therefore, these findings should be re-examined to better understand the molecular mechanism underlying the binding between Cbln4 and their putative partners.

Mutations in cerebellins and their presynaptic neurexin receptors have been associated with multiple neurodevelopmental disorders, especially with autism spectrum disorders (ASDs), Tourette syndrome and schizophrenia (Südhof, 2008; Clarke et al., 2012; Bourgeron, 2015; Pendyala et al., 2017).

### Role of Laminins in Synaptic Organization and Differentiation

Laminins are major components of the basal lamina in different tissues. These glycoproteins consist of α, β and γ chains and organize into cross or T-shaped heterotrimers. Distinct laminin chains assemble in specific regions of different tissues and their expression pattern is dynamic during development. In mammals, five α chains, three β chains and three γ chains have been described. Laminins act as ligands for integrins, dystroglycan, Bcam (basal CAM) and Cav2.1 subunit of presynaptic VGCCs. Laminins have been described to play an active role organizing pre- and postsynaptic structures of the NMJ (Timpl, 1996; Colognato and Yurchenco, 2000; Miner and Yurchenco, 2004; Durbeej, 2010; Rogers and Nishimune, 2017).

Laminin β2 chains assemble with γ1 and either α2, α4 or α5 chains to arrange specific heterotrimers that are integrated into the synaptic basal lamina from skeletal muscle cells (Chiu and Sanes, 1984; Hunter et al., 1989; Martin et al., 1995; Patton et al., 1997; Rogers and Nishimune, 2017). *In vivo* evidence for an essential role of β2 chains at the NMJ comes from β2 KO mouse studies in which it was reported that mice lacking β2 laminin have an inappropriate formation of pre- and postsynaptic terminals leading to malformed NMJs (Noakes et al., 1995; Knight et al., 2003; Chand et al., 2015). In particular, these mice showed a lack of junctional folds, diminished number of active zones and Schwann cell infiltration in the synaptic cleft (Noakes et al., 1995; Patton et al., 1998; Nishimune et al., 2004). Additionally, laminin β2 KO mice have shown to fail in switching from N- to P/Q-type VGCC-mediated transmitter release that normally occurs at presynaptic sites with NMJ maturation (Chand et al., 2015). Also laminin α2, α4 and α5 chains are critical for establishing and maintaining the NMJ structure and alignment of presynaptic active zones (Patton et al., 2001; Nishimune et al., 2008; Holmberg and Durbeej, 2013). Lately, it has been reported that laminin α5 act as a regulator of synapse stability in the CNS during late postnatal development. Conditional deletion of laminin α5 *in vivo* increased dendritic spine size and led to an age-dependent loss of synapses accompanied by behavioral defects. Moreover, it was shown that laminin α5 acts in the brain through an integrin α3β1 adhesion receptor (Omar et al., 2017).

## ECM Role in Synapse Maturation and Plasticity

### Reelin Signaling

Reelin is a large secreted glycoprotein encoded by the *Reln* gene that plays a key role as a regulator of neuronal layering and migration in the cortex, hippocampus and cerebellum during development (Tissir and Goffinet, 2003; Frotscher, 2010; Arcangelo, 2014). Reelin mediates its neuronal guidance action through the binding to the lipoprotein receptors very-low-density lipoprotein receptor (VLDLR) and apolipoprotein E receptor type 2 (APOER2). Activation of reelin signaling pathway, whereby reelin binds to VLDLR and ApoER2, results in cytoplasmic adaptor protein disabled 1 (Dab1) phosphorylation via the Src family of tyrosine kinases (SFK; Beffert et al., 2006; reviewed in Bock and May, 2016; Figure 1B6).

However, once neurons have reached their proper destination, reelin continues to modulate synaptic signaling pathways and regulate synaptic plasticity and axonal and dendritic outgrowth. At this point, inhibitory GABAergic interneurons start to express and secrete reelin (Pesold et al., 1998). This postnatally secreted reelin surrounds spines and dendritic shafts of cortical pyramidal cells (Rodriguez et al., 2000; Pappas et al., 2001). Evidence for the role of reelin regulation of dendritic outgrowth comes from studies in which application of acute and chronic reelin enhanced cortical neuritic outgrowth mobility and size respectively in both wild-type and reelin-deficient neurons. Moreover, they revealed that promotion of outgrowth and stabilization of dendrites by reelin was mediated through an ApoER2/Dab1/PI3K pathway and required activation of mTOR through PI3K and AKT (Leemhuis et al., 2010; Bock and May, 2016; Lee and D’Arcangelo, 2016; Figure 1B6). In agreement with these findings, it was shown that neurons from reelin-deficient mice exhibited diminished dendritic branching and had reduced spine density *in vitro* and *in vivo* (Niu et al., 2004). Niu et al. (2008) showed that ApoER2/VLDLR and both Dab1 and SFKs signal downstream of reelin to regulate spinogenesis and spine morphology (Niu et al., 2008; Bosch et al., 2016; Figure 1B6). Significantly, it was shown that overexpression of ApoER2 in hippocampal neuron cultures increased dendritic spine density, suggesting a crucial role of this receptor in promoting spinogenesis (Dumanis et al., 2011). Reelin has also been shown to support LTP via its binding to postsynaptic ApoER2 and VLDLR. This induces receptor clustering and Dab1 phosphorylation, leading to SFK-mediated tyrosine phosphorylation of the GluN2 NMDAR subunits, which enhances their conductance. Thus, reelin enhances the NMDAR-mediated Ca^2+^ conductance and phosphorylation of cAMP-response element-binding protein (CREB; Chen et al., 2005), leading to a prominent increase in LTP (Weeber et al., 2002; Figure 1B6).

Interestingly, reelin signaling also controls the molecular composition of synapses. During early development, most of the NMDARs at hippocampal synapses consist of GluN2B subunits, which have higher conductance than GluN2A receptors. After maturation of synapses, there is a shift in the NMDARs composition from GluN2B receptors to GluN2A receptors (Cull-Candy et al., 2001; Sinagra et al., 2005). This switch is blocked in cultured hippocampal neurons by inhibiting reelin signaling or its release from GABAergic neurons (Campo et al., 2009). Additionally, it was shown that reelin signaling promotes the transition from “silent” to mature synapses, as it was described that reelin treatment of acute hippocampal slices facilitated the insertion of AMPARs into synaptic membranes containing only NMDARs (Qiu et al., 2006). Furthermore, overexpression of reelin *in vivo* leads to bigger spines and a strong increase in LTP (Pujadas et al., 2010).

Reelin has been shown to be important for both spatial and fear memory. Mice deficient in either ApoER2 or VLDLR have diminished hippocampus-dependent contextual fear memory (Weeber et al., 2002). Reelin function has been associated with several neurological disorders, such as autism spectrum disorders, schizophrenia, depression, bipolar disorder and AD, and impaired and reduced reelin expression seems to be a common characteristic among them (reviewed in Knuesel, 2010; Folsom and Fatemi, 2013; Lane-Donovan et al., 2015).

### Integrin-Dependent Regulation of NMDA Receptors and Synaptic Plasticity

Integrins are a large family of heterodimeric, transmembrane cell surface adhesion receptors present in both the developing and adult brain. They are the major receptors for ECM molecules and are heterodimers composed of α- and β-subunits. During development, integrins modulate cell migration, cortical layer formation, neurite outgrowth and synaptogenesis. In the mature brain, they regulate several synaptic functions (reviewed in Park and Goda, 2016).

β1-containing integrins promote astrocyte-dependent formation of excitatory synapses in hippocampal neuronal cultures (Hama et al., 2004). Additional evidence for the role of β1-containing integrins in synapse regulation comes from *in vivo* work upon conditional deletion of β1 subunits during embryonic stages. Mice with deleted β1-containing integrins showed reduced dendritic arbor size and synapse density in the hippocampal CA1 region at postnatal day 42 (P42), but not at P21, in comparison with their control littermates (Warren et al., 2012). Conversely, when ablation of β1-containing integrins was induced at later stages (P14), no evidence of structural irregularities was observed in adult mice, although a functional deficit in synaptic plasticity was found (Huang et al., 2006). Interestingly, analogous late-appearing phenotype of diminished dendrite arbor size and synapse density was revealed in Abl2/Arg kinase-deficient mice (Sfakianos et al., 2007) and in mice in which the α3 subunit was conditionally deleted (Kerrisk et al., 2013), suggesting that integrin-regulated Abl2/Arg kinase may act downstream of α3β1 integrin heterodimers.

Like reelin, integrin binding partners might support the induction of LTP via modulation of NMDARs. Indeed, β3-containing integrins and integrin-regulated Abl2/Arg kinase have shown to support synapse maturation by inducing a switch in postsynaptic NMDAR subunits from GluN2B-containing to GluN2A-containing receptors and to induce the developmental reduction in presynaptic neurotransmitter-release efficacy, which are common features of mature synapses (Chavis and Westbrook, 2001; Xiao et al., 2016). β3-containing integrins have shown to play a key role in regulating AMPARs at excitatory synapses (Figure 1A3). Interfering with β3-containing integrins ligation to the ECM either by RGD peptides or by overexpressing a dominant-negative form of the β3 subunit decreased synaptic strength by reducing the number of postsynaptic AMPARs. Moreover, these effects on AMPAR stabilization require basal NMDAR activity, Ca^2+^ influx and activation of the small GTPase Ras-related protein Rap1 (Cingolani et al., 2008).

Interestingly, in cultured spinal cord neurons, β1- and β3-containing integrins have shown to regulate the synaptic strength by controlling the trapping of glycine receptors at inhibitory synapses. Particularly, β1-containing integrins showed to increase synaptic glycine receptor abundance, whereas β3-containing integrins decreased it. Interestingly, these effects were similar to those of TSP1 and fibrinogen, which are ligands for β1- and β3-containing integrins, respectively. Considering that TSP1 and fibrinogen are released after injury, integrins may play a key role in adjusting neuronal excitability under pathological conditions (Charrier et al., 2010).

β3-containing integrins are necessary for homeostatic synaptic plasticity, a process by which synaptic strength is regulated by adjusting the functional availability of AMPARs at postsynaptic sites. In hippocampal cultures, persistent block of action potentials with tetrodotoxin leads to a “scaling up” (upregulation) of the AMPARs present at the synapses proportionally to their synaptic expression before the treatment. Meanwhile, β3-subunit expression at the surface also increases. In agreement with these observations, tetrodotoxin-induced scaling up of AMPARs was absent in hippocampal cultures from β3-subunit-deficient mice (Cingolani and Goda, 2008; Cingolani et al., 2008). Significantly, tumor necrosis factor alpha (TNFα) proved to increase surface expression of β3-containing integrins in hippocampal neurons (Cingolani et al., 2008) and proved to be necessary for tetrodotoxin-induced synaptic scaling up of AMPARs (Stellwagen and Malenka, 2006). Thus, TNFα and β3-containing integrins may participate in the signaling pathway that mediates homeostatic AMPAR scaling.

The role of integrins in LTP was originally studied using RGD peptides and blocking-antibodies against integrins. In acute hippocampal slices, high concentrations of RGD peptides impaired maintenance of LTP in CA1 area. However, induction of LTP appeared to be intact. Similarly, inhibitory antibodies against α5- and β1-containing integrins decreased LTP stabilization in CA1 region. Later, *in vivo* studies using mouse models elucidated that α3-, α5-, α8- and β1-containing integrins were important for LTP maintenance (reviewed in Park and Goda, 2016). Moreover, many studies described that β1-containing integrins regulate NMDARs. In fact, RGD or fibronectin administration rapidly phosphorylated FAKs and SFKs. SFK activation increased tyrosine phosphorylation of GluN2A and GluN2B NMDAR subunits, which enhanced NMDAR activity (Bernard-Trifilo et al., 2005; Chen and Roche, 2007).

Dysfunctions on integrins signaling have been associated extensively with several neuropsychiatric disorders and neurodegenerative diseases such as anxiety, stress, autism spectrum disorder, drug addiction and AD (reviewed in Park and Goda, 2016). However, many gaps remain to be filled to understand integrin-mediated mechanisms and functions at all levels.

### Tenascins and Synaptic Plasticity

Multiple ECM molecules have been discovered to play a role in synaptic plasticity (reviewed by Senkov et al., 2014). Here, we focus on tenascins as a family of large glycoproteins, which were first among ECM molecules demonstrated to modulate synaptic plasticity and for which underlying mechanisms were dissected. Tenascins interact with other ECM molecules and receptors through their tenascin’s EGF-like domain and fibronectin type III-repeats. Expression of tenascins is dynamic during development and regulated by diverse molecules like growth factors, cytokines, vasoactive peptides and ECM proteins (Jones and Jones, 2000; Senkov et al., 2014; Heck and Benavides-Piccione, 2015). Tenascin-R (TNR) and tenascin-C (TNC) are predominantly expressed in the CNS and represent the most studied members of tenascin family in terms of synaptic plasticity. Both TNR and TNC have been implicated in functions like myelination and axonal growth during the developmental period and in hippocampal synaptic plasticity in the mature brain (Dityatev and Schachner, 2003; Dityatev et al., 2010). TNR is expressed by oligodendrocytes, Purkinje cells, motor neurons and some subsets of interneurons (Dityatev and Schachner, 2003). TNR is one of the major components in the PNNs (Dityatev and Schachner, 2003; Suttkus et al., 2014). TNR is necessary for normal synaptic plasticity, synaptic transmission and behavior. TNR-deficient mice showed abnormal structure of PNNs, impaired LTP but normal LTD in CA1 area of the hippocampus, increased basal synaptic transmission, and anxiety and motor impairments (Bukalo et al., 2001; Saghatelyan et al., 2001; Freitag et al., 2003; Gurevicius et al., 2004). TNR has been shown to carry the human natural killer 1 (HNK1) carbohydrate epitope which has been shown to inhibit postsynaptic GABA_B_ receptors. Interference with HNK1 glycan leads to increased GABA_B_ receptors activity and impairs evoked release in perisomatic GABAergic synapses on CA1 pyramidal cell layer (Saghatelyan et al., 2001, 2003). Moreover, TNR-deficient mice exhibited a strong reduction in the number of active zones in perisomatic inhibitory synapses in CA1 pyramidal cell layer compared with wild-type mice, suggesting that TNR may play a crucial role in the regulation of the architecture of perisomatic inhibitory synapses (Nikonenko et al., 2003). Furthermore, Bukalo et al. (2007) reported that application of HNK1 glycomimetic or pharmacological treatment with either GABA_A_ receptor agonist, a GABA_B_ receptor antagonist, an L-type VGCC blocker or an inhibitor of protein serine/threonine phosphatases restored LTP from TNR KO mice slices to the levels seen in wild-type mice. These observations suggested that a chain of events initiated by impaired GABAergic transmission and proceeding via Ca^2+^ entry into cells and elevated activity of phosphatases mediates metaplastic adjustment of hippocampal plasticity in the absence of TNR (Bukalo et al., 2007).

TNC is highly expressed in the developmental period but decreases through adolescence and their levels are very low in adults (Ferhat et al., 1996). However, it has been shown that LTP induces transient TNC expression in mature brain, proposing that TNC can play a crucial role regulating synaptic plasticity (Nakic et al., 1998). Indeed, it was later found that TNC-deficient mice exhibited a reduction in L-type VGCC-dependent forms of LTP and abolished LTD in CA1 hippocampal region (Evers et al., 2002; Strekalova et al., 2002). Interestingly, the L-VGCC activator Bay K-8644 rescued LTP impairment in this region (Morellini et al., 2017). TNC isoforms containing repeat FNIII A3 are susceptible to proteolytic degradation by MMP2 and 3 and ablation of MMP3 also results in specific impairment of L-type VGCC-dependent LTP (Wiera et al., 2017). Also, enzymatic digestion of hyaluronic acid with hyaluronidase has a similar effect (Kochlamazashvili et al., 2010; Figure 1B7), suggesting that a complex of ECM molecules and their proteolysis are involved in regulation of L-type VGCC activity. *In vivo*, it was reported that the power of theta and gamma oscillations was increased in cortex and hippocampus of TNC-deficient mice in comparison to wild-type mice. Interestingly, gamma rhythm was specifically enhanced in the CA1 region but not in other hippocampal areas of these mutants. Furthermore, morphological analyses showed reduction in CA1 volume and decreased number of somatostatin-positive interneurons in the hippocampus. Recently, it has been shown that TNC-deficient mice have normal learning and memory in the contextual fear conditioning paradigm but impaired extinction of conditioned fear responses. In agreement with these observations, TNC deficiency mimicked and occluded the effects of systemic administration of the L-VGCC blockers nifedipine and diltiazem on fear extinction (Morellini et al., 2017).

Overall, these findings suggest a role for TNC in structural organization and in shaping neural activity in the hippocampus, particularly in the CA1 area and that TNC-mediated modulation of L-VGCC activity is essential for fear extinction (Gurevicius et al., 2009; Morellini et al., 2017).

## Activity-Dependent ECM Remodeling

Extracellular proteolysis at the synapse has been recognized to play a key role in synaptic plasticity and determining dendritic spine shape and function and thus regulating learning and memory functions. Particularly, extracellular proteases regulate structural modification of synapses through different pathways. These include proteolysis of ECM, synaptically expressed CAMs and neurotrophic factors (Sonderegger and Matsumoto-Miyai, 2014; Wójtowicz et al., 2015). In this part of the review we highlight possible importance of activity-dependent concerted activation of multiple extracellular proteases, such as ADAMTS4/5, MMP9 and neurotrypsin, for permissive and instructive events in synaptic remodeling through the cleavage of perisynaptic ECM and generation of proteolytic fragments as inducers of synaptic modifications.

### Matrix Metalloproteinases in the CNS

MMPs form a large subgroup of zinc-binding, multidomain endopeptidases that are expressed in most tissues of the body. They belong to the bigger metzincin family of metalloproteinases, named for the conserved methionine residue close to the zinc ion-dependent metalloproteinase active site (Gomiz-Rüth, 2009). In humans, 23 MMPs members have been found, including secreted and transmembrane proteins. In the CNS, MMPs are synthesized and secreted by neurons and glia. The majority of MMPs are synthesized and secreted in a zymogen form as inactive pro-enzymes that are later converted to proteolytically active enzymes after several regulatory steps (reviewed in Huntley, 2012; Stawarski et al., 2014). Active MMPs can target, modify and proteolytically process ECM components and consequently control cell behavior (Sternlicht and Werb, 2001; Butler and Overall, 2009). TIMPs are inhibitors of MMPs function, acting in synergy with MMPs to restrain extracellular proteolysis in space and time (Murphy, 2011; Arpino et al., 2015). MMP-mediated extracellular remodeling in the brain can dually act regulating cell behavior, as it can have a permissive (degrading chemical or physical barriers) and instructive role (initiating or terminating signaling cascades through the processing of molecules) to establish persistent modifications in both synapse structure and function (Sternlicht and Werb, 2001; Butler and Overall, 2009). In mouse and rat brains, high levels of the pro-forms of MMP2, 3, 9 and 24, but lower levels of the active forms of these MMPs were found in in hippocampal lysates (Szklarczyk et al., 2002; Bozdagi et al., 2007; Huntley, 2012). High-resolution imaging of hippocampal tissue sections revealed that MMP9 is concentrated at synaptic puncta and colocalizes with pre- and postsynaptic markers (Bozdagi et al., 2007). Likewise, MMP24 showed a punctate pattern expression and synaptic-like distribution in mouse hippocampus and cerebellum (Sekine-Aizawa et al., 2001). It colocalizes with synaptic markers in cultured rat hippocampal neurons (Restituito et al., 2011). Moreover, both MMP9 and MMP24 colocalize with excitatory synaptic markers, including AMPARs and NMDARs, scaffolding and cytoskeletal proteins and presynaptic vesicle proteins (Bozdagi et al., 2007; Wilczynski et al., 2008). On the contrary, their expression is absent in GABAergic inhibitory synapses (Wilczynski et al., 2008).

Our current understanding on how MMPs contribute to synaptic plasticity and synapse remodeling comes mostly from extensive studies of MMP9. Nagy et al. (2006) and Bozdagi et al. (2007) showed that levels of both the pro-form and the active form of MMP9 were increased after the induction of late-phase LTP (L-LTP) in the CA1 area of hippocampus. Moreover, they evinced that MMP-9 inhibition led to a rapid return of synaptic potentiation to baseline levels, indicating that upregulation of MMP9 is mechanistically related to L-LTP (Nagy et al., 2006; Wang et al., 2008). MMP9 knock-out mice showed impaired LTP that could be restored by applying proteolytically active MMP9 fragments (Nagy et al., 2006). Several studies demonstrated a role of MMP9 in elongation and spine thinning, although MMP9 action on dendritic spines is not fully understood (Wang et al., 2008; Bilousova et al., 2009; Michaluk et al., 2011; Kondratiuk et al., 2017). Michaluk et al. (2011) showed that overexpression of activated MMP9 and application of active MMP9 in both hippocampal cultures and organotypic slices induced dendritic spine elongation (Michaluk et al., 2011). Additional evidence for the contribution of MMP9 to the spine elongation that accompanies LTP stabilization comes from combination of two-photon dendritic spines imaging and whole-cell recordings from hippocampal neurons. Wang et al. (2008) found that MMP9 effects on spine enlargement and synaptic potentiation were mediated through β1-containing integrin receptors and were associated with integrin-dependent phosphorylation (and thus inactivation) of the actin-depolymerizing component cofilin within spines. Interestingly, MMP inhibitors have no effects on baseline properties of synaptic neurotransmission and spine size and morphology, suggesting that MMPs specifically act on regulating synaptic remodeling following induction of LTP or acquisition of memories. In agreement with this finding, hippocampal baseline neurotransmission, magnitude or time course of paired-pulse facilitation (PPF), which is a form of presynaptic plasticity or LTD were not affected in MMP9-deficient mice (Nagy et al., 2006). Recently, it was also described that MMP9 contributes to ECM degradation, alters synaptic dynamics and sensory-evoked plasticity during ocular dominance plasticity in the mouse visual cortex (Kelly et al., 2015). Similar roles of MMP9 in synaptic and spine plasticity have been described in other brain regions, including the central amygdala (Stefaniuk et al., 2017), and the prefrontal cortex (Okulski et al., 2007). There are several identified and proposed molecules to be potential MMP targets and therefore to contribute to synaptic remodeling via MMP-mediated proteolysis. One of them is the intercellular cell adhesion molecule 5 (ICAM5). It has been proposed that full length ICAM5 is expressed in immature neurons and cleaved by MMPs and its cleavage releases an N-terminal extracellular domain which is detectable 15 min after LTP induction. Moreover, soluble ICAM5 increases AMPAR expression and cofilin phosphorylation (Conant et al., 2011; Lonskaya et al., 2013). Additionally, ICAM5 soluble extracellular domain has shown to promote dendritic filopodia elongation (Tian et al., 2007). Notably, there is a shift in ICAM5 localization in cortical neurons during synapse development from dendritic spines and filopodia to the dendritic shaft, and this shift is not observed in MMP9-deficient mice (Kelly et al., 2014), suggesting that ICAM5 is a crucial MMP9 substrate during spinogenesis and synaptogenesis. More recently, CD44—a major ECM receptor for hyaluronic acid—was reported to be cleaved by MMP9 in response to stimulation of 5-HT7 receptors (Bijata et al., 2017). This signaling activated the small GTPase cdc42 and promoted neuronal outgrowth and elongation of dendritic spines.

Beyond its role in physiological synaptic plasticity and spine remodeling, MMP9 plays a role in several pathologies (Rivera et al., 2010; Kaczmarek, 2013; Reinhard et al., 2015; Vafadari et al., 2016; Lepeta et al., 2017). Indeed, research on MMP9 was at first focused on its role in the CNS pathology such as post-injury and post-stroke damage in brain tissue due to its proteolytic activity. Future studies will be necessary to identify all MMP targets and elucidate the detailed mechanism of MMP-mediate proteolysis in inducing persistent modifications in both synapse structure and function.

### The ADAMTS Family

The ADAMTS proteases belong to “a disintegrin and metalloproteinase with TSP motifs” family, comprising 19 members (Tang, 2001; Apte, 2004). They are members from the metzincin protease superfamily. ADAMTS proteinases are synthesized as pre-pro-enzymes. Pre-pro-ADAMTS proteases can be cleaved at both C- and N-terminal fragments by furin or pro-protein convertases resulting in secretion of mature and potentially active enzymes lacking the propeptide domain (Flannery et al., 2002; Lemarchant et al., 2013). ADAMTS contain an ancillary C-terminal domain responsible for their interactions and associations with the ECM, regulation of their activity and specification of their binding partners’ interactions. This domain contains a 50-amino acid TSP-like repeat sequence that it is similar and shared with TSPs 1 and 2 (Adams and Lawler, 2011). ADAMTS function is endogenously inhibited and regulated by TIMPs (Murphy, 2011). The ADAMTS family members are classified in distinct subgroups according to their preference to cleave specific ECM molecules. They are involved in several functions including anti-angiogenesis processes, maturation of collagen fibrils, blood coagulation and recovery and repair following spinal cord injury (Gottschall and Howell, 2015; Kelwick et al., 2015). Among ADAMTS members expressed in the brain are ADAMTS1, 4, 5, 8, 9 and 15, which have the ability to cleave lecticans, including aggrecan, brevican, versican and neurocan. However, lecticans are not the only substrate of this ADAMTS subfamily as they have shown to cleave also phosphacan (Tauchi et al., 2012) and reelin (Hisanaga et al., 2012; Krstic et al., 2012). *In vivo* data supports the view that most ADAMTSs are produced by astrocytes, especially after injury, although ADAMTSs are also produced by neurons and microglia (Lemarchant et al., 2013).

The first *in vivo* evidence for a role of ADAMTS1 and 4 in ADAMTS-dependent cleavage of brevican comes from Yuan et al. (2002) as they found that after kainate-induced CNS lesion, ADAMTS-induced cleavage of brevican was stimulated and this was associated with reduced synaptic density in the dentate gyrus. In agreement with this finding, it was later found that ADAMTS4 induced neurite elongation *in vitro* (Hamel et al., 2009). On the other hand, after localized entorhinal cortex lesion, synaptic density in the molecular layer of the dentate gyrus (measured by synaptophysin levels) was significantly reduced at day 2 and 7 post injury, but not at day 30. In the same way, brevican expression was elevated at day 2 and 7 but returned to basal levels at day 30. However, when ADAMTS activity was evaluated, data showed a significant increase at day 7 but not at day 2 or 30, suggesting that ADAMTS activity is elevated during the initial synaptic re-innervation period (7 days after lesion) and may modulate the process of sprouting and/or synaptogenesis (Mayer et al., 2005).

Furthermore, another *in vivo* study reported that the expression of several synaptic markers including synaptosome-associated protein 25 (SNAP-25), synaptophysin and postsynaptic density protein 95 (PSD-95), was significantly lower in female ADAMTS1 null frontal cortex, but not in male mice, suggesting a gender dimorphism of ADAMTS1 involvement in synaptic plasticity. Nevertheless, the reduction in synaptic proteins expression was not accompanied by deficits in learning and memory (Howell et al., 2012). ADAMTS4 and ADAMTS5 have been shown to cleave reelin and their expression levels and localization correlated with those of reelin in the mouse hippocampus. Interestingly, ADAMTS5 protein levels are dramatically reduced in aged 3xTg-AD AD mouse model. Taken into account that ADAMTS5 is expressed in dendrites of the hippocampal pyramidal neurons, it could be reasonable that reelin aggregation in the stratum radiatum of AD mice would be, at least in part, due to the diminished reelin degradation by ADAMTS5 (Krstic et al., 2012).

Overall, the majority of data suggests that ADAMTS proteoglycanases are upregulated in response to CNS injury and disease as they may be involved in the activation of plasticity mechanisms on neurites and synapses.

### Neurotrypsin: A Serine Protease Involved in Spinogenesis

The neuronal serine protease neurotrypsin (also called motopsin or Prss12) has been recognized to play an important role in cognitive processes in humans. A 4-nucleotide deletion in the coding region of the protein, that results in the expression of a truncated version of the protease lacking the catalytic domain, has been associated with severe forms of non-syndromic mental retardation (Molinari et al., 2002). Neurotrypsin is predominantly expressed in neurons of the cerebral cortex, the hippocampus and the amygdala (Gschwend et al., 1997; Wolfer et al., 2001), regions that are involved in the processing of learned behaviors and formation of new memories. In the CNS, neurotrypsin is stored in presynaptic terminals, in the region lining the synaptic cleft of both excitatory and inhibitory synapses (Molinari et al., 2002; Stephan et al., 2008). Interestingly, live imaging studies on cultured hippocampal neurons elucidated that neurotrypsin is released from presynaptic boutons in an activity dependent manner (Figure 1B5). Particularly, they showed that once externalized, neurotrypsin remains at the synapse for several minutes before it disappears, suggesting a transient binding of neurotrypsin to a surface receptor or ECM protein (Frischknecht et al., 2008). Indeed, the unique neurotrypsin target in the CNS is the heparin sulfate proteoglycan agrin (Reif et al., 2007). Agrin has been comprehensively studied for its role as a crucial organizer of postsynaptic differentiation at the NMJ (Bezakova and Ruegg, 2003). At the NMJ, agrin acts via MuSK (Glass et al., 1996), a transmembrane tyrosine kinase and its co-receptor LRP4, a LDLR-related protein (Kim et al., 2008; Zhang et al., 2008). However, later, agrin showed to be involved in the formation of CNS synapses. Here, agrin signaling is mediated via binding and inhibiting the α3 subunit of the Na+/K+ ATPase (α3NKA; Hilgenberg et al., 2006). Synaptic agrin is cleaved by neurotrypsin at two homologous and highly conserved C-terminal sites, releasing a 90-kDa (agrin 90) and a 22-kDa (agrin 22) fragment (Figure 1B5). Both agrin fragments are lacking in the brain of neurotrypsin-deficient mice, indicating that cleavage of agrin in the brain depends exclusively on neurotrypsin in the CNS (Reif et al., 2007, 2008). Intriguingly, Matsumoto-Miyai et al. (2009) demonstrated that agrin cleavage requires not only neurotrypsin exocytosis after presynaptic depolarization but also depends on concomitant postsynaptic activity. They found *in vitro* that blockage of NMDAR with MK801 inhibited the neurotrypsin-dependent agrin cleavage without affecting neurotrypsin secretion from presynaptic terminals, suggesting that neurotrypsin is secreted in its inactive form and that NMDA receptor-driven activity of the postsynaptic cell is required for its activation (Matsumoto-Miyai et al., 2009). However, the mechanism of neurotrypsin activation is still unknown, although it has been reported that a zymogen activation site at the N-terminal of neurotrypsin bears the proprotein convertase (PC) recognition sequence RRQKR (Reif et al., 2008; Figure 1B5).

Interestingly, it was found that LTP was intact in hippocampal slices from neurotrypsin-deficient mice. However, LTP-associated formation of filopodia was abolished in these conditions but could be completely rescued by exogenous administration of agrin 22 (Matsumoto-Miyai et al., 2009). Considering that dendritic filopodia are thought to be precursors of synapses and neurotrypsin-dependent agrin cleavage requires coincident pre- and postsynaptic activation, these results imply neurotrypsin-dependent agrin cleavage to be involved in Hebbian learning and remodeling of synaptic circuits in the CNS.

Additional evidence for the potential role of neurotrypsin in synaptic homeostasis comes from a recent study in which it was postulated that synaptic dysfunction found in Cln1−/− mice, a mouse model of infantile neuronal ceroid lipofuscinosis (INCL), could be due to, at least in part, a novel mechanism that links oxidative stress with suppression of agrin 22 production in INCL disease. Interestingly, they showed that treatment with a specific antioxidant, NtBuHA, elevated agrin 22 levels, and therefore, may have therapeutic implications for this devastating disease (Peng et al., 2015).

## Concluding Remarks

Cognitive functions, including learning and memory, depend on adaptive changes either in the efficacy of signal transmission of existing synapses, and/or structural modification, addition or elimination of connections between pre- and postsynaptic neurons. Over the last two decades, evidence has been accumulated that activity-dependent aggregation and proteolysis of ECM and associated molecules shapes synaptogenesis, synapse maturation and synaptic circuit remodeling. In this review, we have highlighted some of molecular mechanisms by which ECM is involved in both structural and functional synaptic plasticity and maintains synaptic homeostasis at the same time (Figure 1). However, functions of many ECM ligands and molecular mechanisms underlying the interactions between ECM and their targets remain still unknown. Future studies in this field are warrantied to identify key ECM aberrations associated with synaptopathies in major neurological and psychiatric disorders and to target these mechanisms therapeutically to achieve a control over synaptic flexibility and stability.

## Author Contributions

AD developed the concept. MF-F has written a draft, both edited the text.

## Conflict of Interest Statement

The authors declare that the research was conducted in the absence of any commercial or financial relationships that could be construed as a potential conflict of interest.
